# Dietary Flavonoids as Cancer Chemopreventive Agents: An Updated Review of Human Studies

**DOI:** 10.3390/antiox8050137

**Published:** 2019-05-18

**Authors:** Carmen Rodríguez-García, Cristina Sánchez-Quesada, José J. Gaforio

**Affiliations:** 1 Center for Advanced Studies in Olive Grove and Olive Oils, University of Jaen, Campus las Lagunillas s/n, 23071 Jaén, Spain; crgarcia@ujaen.es (C.R.-G.); csquesad@ujaen.es (C.S.-Q.); 2 Department of Health Sciences, Faculty of Experimental Sciences, University of Jaén, 23071 Jaén, Spain; 3 Agri-Food Campus of International Excellence (ceiA3), 14005 Córdoba, Spain; 4 CIBER Epidemiología y Salud Pública (CIBER-ESP), Instituto de Salud Carlos III, 28029 Madrid, Spain

**Keywords:** flavonoids, diet, antioxidants, cancer

## Abstract

Over the past few years, interest in health research has increased, making improved health a global goal for 2030. The purpose of such research is to ensure healthy lives and promote wellbeing across individuals of all ages. It has been shown that nutrition plays a key role in the prevention of some chronic diseases such as obesity, cardiovascular disease, diabetes, and cancer. One of the aspects that characterises a healthy diet is a high intake of vegetables and fruits, as both are flavonoid-rich foods. Flavonoids are one of the main subclasses of dietary polyphenols and possess strong antioxidant activity and anti-carcinogenic properties. Moreover, some population-based studies have described a relationship between cancer risk and dietary flavonoid intake. In this context, the goal of this review was to provide an updated evaluation of the association between the risk of different types of cancers and dietary flavonoid intake. We analysed all relevant epidemiological studies from January 2008 to March 2019 using the PUBMED and Web of Science databases. In summary, this review concludes that dietary flavonoid intake is associated with a reduced risk of different types of cancer, such as gastric, breast, prostate, and colorectal cancers.

## 1. Introduction

Cancer is among the diseases that have the greatest impact on society [1]. Even though its incidence has increased over the years, its mortality has decreased because of advances in treatment [2]. However, efforts to improve cancer prevention are needed. The aetiology of cancer is multifactorial, involving both environmental and genetic factors [3]. Diet is one of the lifestyle factors that affect cancer incidence and mortality [4]. Recently, several studies have reported that diets based on high levels of vegetables and fruits are strongly associated with a significant reduction in cancer risk [5,6].

Furthermore, there are some bioactive compounds in foods that have potential health benefits, such as flavonoids, carotenoids, stilbenes, lignans, and phenolic acids [7,8]. Flavonoids are a large group of phenolic compounds and are usually involved in protection against harsh environmental conditions, UV radiation, and microorganism attacks in plants [9,10]. Because of their potent antioxidant activity against oxidative stress, the interest in flavonoids has recently increased [11]. In vitro and in vivo studies have demonstrated that they have anti-carcinogenic properties against different types of cancers [5,12]. Moreover, many population-based studies have described an association between dietary flavonoids and cancer risk [13,14]. Hence, the goal of this review is to perform an updated evaluation of the association between the risk of different types of cancers and dietary flavonoids, as well as the intake of each flavonoid subclass.

## 2. Methodology

Recently, interest in flavonoids has increased because their strong antioxidant and anti-carcinogenic activities may have possible beneficial effects on cancer. Thus, in this review, we analysed all relevant cancer epidemiological studies from January 2008 to March 2019 using the PUBMED and Web of Science databases [15,16]. Since different reviews have already been published on flavonoids and cancer before 2008. Search entries included [flavonoids and cancer], [flavonoids and “breast cancer”], [flavonoids and “lung cancer”], [flavonoids and “prostate cancer”], [flavonoids and “gastric cancer”], [flavonoids and “pancreatic cancer”] and [flavonoids and “colorectal cancer”]. Selection criteria applied were the following: human studies, randomized controlled trials, cross-sectional, cohort and case-control studies and information about dietary intake. Reviews studies and Meta-Analyses were excluded. Besides, population studies were catalogued based on type of study: case-control or cohort study and the type of cancer.

## 3. Biosynthesis and Subclasses of Flavonoids

**Figure 1:**
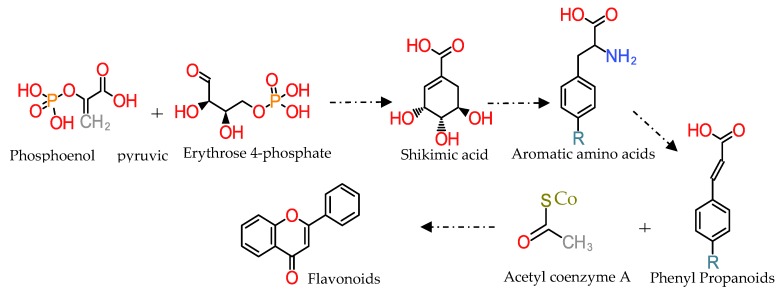
Flavonoid biosynthesis pathways (general structure of flavonoids).

Flavonoids are secondary metabolites synthesised mainly by plants [9]. To date, more than 6000 different flavonoids have been identified, and they are distributed in a wide range of plants [17]. The general structure of flavonoids is composed of a 15-carbon skeleton, containing 2 benzene rings connected by a 3-carbon linking chain (Figure 1) [9]. Therefore, they are depicted as C6-C3-C6 compounds. Their biosynthesis involves two different biosynthetic pathways: the shikimic acid pathway and the acetate pathway (Figure 1) [9].

**Figure 2:**
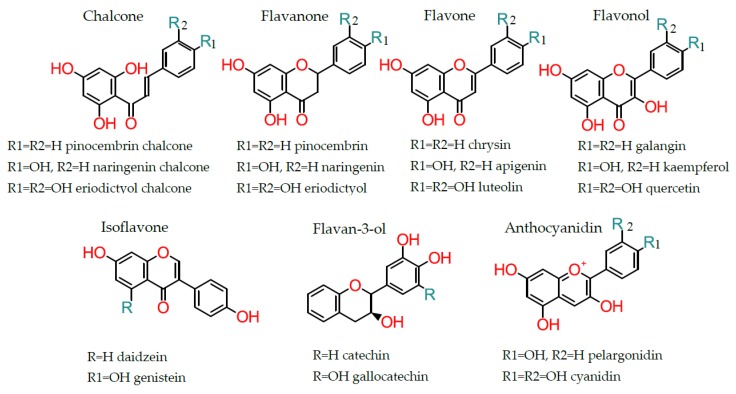
Flavonoid subclasses.

Depending on the chemical structure, degree of oxidation, and unsaturation of the linking chain (C3), flavonoids can be classified into different groups, such as anthocyanidins, chalcones, flavonols, flavanones, flavan-3-ols, flavanonols, flavones, and isoflavonoids (Figure 2). Furthermore, flavonoids can be found in plants in glycoside-bound and free aglycone forms [9]. The glycoside-bound form is the most common flavone and flavonol form consumed in the diet [9].

## 4. Dietary Flavonoids

Flavonoids are widely spread in different foods and beverages (such wine and tea), but the sources with the highest levels are fruits and vegetables [10]. Among the fruits (Table 1), the highest levels of flavonoids are found in berries, such as black elderberry (1358.66 mg/100 g) and black chokeberry (1012.98 mg/100 g) [18,19]. In the drupes group, some fruits such as plum and sweet cherry have higher levels of flavonoids than the rest of the group, 101.67 mg/100 g and 185.05 mg/100 g, respectively [20,21]. In the pomes group, apple has the level (56.35 mg/100 g) [21,22]. Furthermore, tropical fruits have a very low flavonoid content [23]. Depending on the type of fruit, the main flavonoid subclass groups vary: anthocyanins predominate in berries, and flavanols predominate in pomes, tropical fruits, and drupes (except in sweet cherry).

Regarding vegetables (Table 2), the foods with the highest levels of flavonoids are broad bean pod (189.54 mg/100 g) [25], black olive (159.83 mg/100 g) [26], red onion (131.51 mg/100 g) [27], spinach (119.27 mg/100 g), and shallot (112.22 mg/100 g) [28,29]. Except for broad bean pod, the predominate flavonoid subclass in vegetables is flavanols.

Regarding seeds (Table 3), although common bean has high levels of flavonoids (from anthocyanins and flavonols), the foods with the highest levels are those derived from soy, and soy products have been suggested to play a key role in the prevention of different diseases [30].

Regarding cereals (Table 4), some such as barley, buckwheat, and common wheat contain average levels of flavonoids (35.2 mg/100 g, 37.04 mg/100 g, and 77.4 mg/100 g, respectively). However, it is important to note that the highest levels are found in whole grains, and levels are greatly reduced when grains are heat treated or refined [30,31].

Cocoa and its products, such as dark and milk chocolate, are flavonoid-rich foods (Table 5). In these foods, the main flavonoids are flavanols, with cocoa containing 511.63 mg/100 g [32,33].

Regarding oils, the data collected from the Phenol Explorer database refer only to oils made from olives (Table 6). In ascending order, refined, virgin, and extra virgin olive oil contain 0.15 mg, 0.23 mg, and 1.53 mg of flavones in 100 g, respectively [34,35].

For beverages, a distinction can be made between non-alcoholic (Table 7) and alcoholic drinks (Table 8). The non-alcoholic drinks with the highest levels of flavonoids are tea infusions, particularly black (83.35 mg/100 g) and green tea (77.44 mg/100 g), and these are mainly flavanols [36,37]. The second most flavonoid-rich beverages are fruit juices, notably pure apple juice (54.99 mg/100 g), pure orange juice (48.02 mg/100 g), pure grapefruit juice (47.12 mg/100 g), and pure lemon juice (37.43 mg/100 g) [38]. The main flavonoids in citrus juices and grapefruit juice are flavanones [39]. However, the main flavonoids in pome juices are flavanols. Regarding alcoholic beverages, wine red contains the highest flavonoid level (83.96 mg/100 mL) [40,41].

Therefore, a diet rich in fruits, vegetables, seeds, and cereals will provide large amounts of flavonoids. However, it is important to know that there are some foods which contain high quantities of flavonoids, including berries, black olives, spinach, onions, soy products, cocoa, whole grain cereals, tea infusions, and red wine.

## 5. Pharmacokinetics

In order to determine the biological activity and physiological functions of flavonoids *in vivo*, their bioavailability must be known. Hence, it is necessary to understand the processes of absorption, digestion, metabolism, and excretion in the digestive tract.

**Figure 3:**
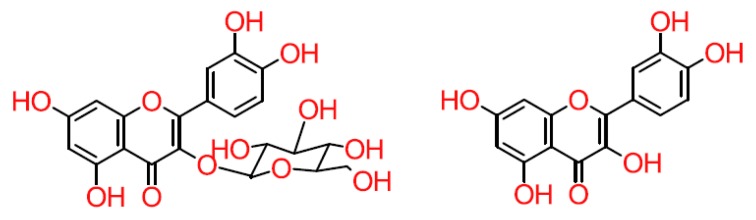
Structure of glycoside and aglycone flavonoids.

Although dietary flavonoids are mostly found in their glucoside form (Figure 3), they are not found in plasma [42,43] because, once flavonoids enter the oral cavity, they begin to be hydrolysed [42]. In addition, their absorption throughout the digestive tract is associated with the hydrolysing activity of different enzymes [44]. In the small intestine, deglycosylation occurs in which two enzymes that act as β-glucosidases are involved: lactase-phlorizin hydrolase (LPH) and cytosolic β-glucosidase (CBG), which are located in the brush border of epithelial cells and enterocytes, respectively [42,45]. Flavonoid-O-β-D-glucosides, for which LPH has high specificity, can enter into cells by passive diffusion. However, glucosides enter enterocytes via sodium-glucose co-transporter type 1 (SGLT1)) [42,44,46]. Although β-glucosidases cannot hydrolyse non-monoglucosidic glycosides, gut microbiota compensate for this through the production of absorbable aglycon in the large intestine and cecum (Figure 3) [42].

Once flavonoids and aglycons are absorbed via the small and the large intestine, respectively, the second phase of enzymatic metabolism begins [42,44]. In this stage, three types of enzymes are involved (uridine-5ʹ-diphosphate-glucuronosyltransferases, sulfotransferases, and catechol-O-methyltransferases) that can conjugate flavonoids with glucuronic acid, sulphate, and methyl groups, making them more water-soluble [13,47]. This phase begins in the wall of the small intestine where metabolites pass to the portal vein and are transported to the liver. In the liver, metabolites are conjugated by sulphation and methylation processes [42]. In the systemic circulation and urine, there are different chemical forms of flavonoids. However, in human plasma, aglycons are rarely detected [42,48,49,50]. Certain plasmatic metabolites are usually excreted into the intestine through bile, and here, they are deconjugated by microbiota and reabsorbed [42,51]. Thus, enterohepatic circulation increases the half-life of flavonoids in human plasma [40].

The gut microbiome plays a main role in the metabolism and absorption of flavonoids. However, these processes could be modified due to flavonoids interaction with other nutrients [52,53]. Among them, flavonoids could alter glucose absorption after high carbohydrate food intake, because inhibit carbohydrate-hydrolyzing enzymes (α-amylase and α-glucosidase) [54]. Besides, flavonoids inhibit glucose transporter in the brush border [54]. However, flavonoid bioavailability is modified with fats intake that improves flavonoid intestinal absorption due to the increment of bile salts secretion which enhances micellar incorporation of flavonoids [54]. However, regarding proteins intake, flavonoid bioavailability became worse [55]. It has been demonstrated that the interaction of phenolic acids with proteins affects antioxidant efficacy and protein digestibility [56].

Depending on the type of flavonoid and its source, bioavailability may differ. Quercetin is one of the most frequently consumed flavonoids (the main sources of quercetin are onions, apples, tea, and wine), being mainly found in its glycosylated form [13]. For example, quercetin glycosides from apples have lower bioavailability than those from onions [13,57]. The plasma levels of quercetin metabolites range from 0.7 to 7.6 µM [13].

Other studies have analysed the levels of flavonoids in human plasma after the intake of flavonoid-rich foods [13]. They could be grouped according to the flavonoid subclass. Flavonols present in apples, onions, and buckwheat tea are found after intake at plasma levels of 0.30 µM, 0.74–7.60 µM, and 2.10 µM, respectively [13,57]. For flavanols in red wine, black tea, green tea, and cocoa, the plasmatic concentration after intake is around 0.08 µM, 0.09–0.34 µM, 1.00–1.80 µM, and 4.92–5.92 µM, respectively [13,58,59,60]. The base plasma levels of flavanones in orange juice and grapefruit juice are around 0.06–0.64 µM and 5.99 µM, respectively, after intake [61]. Finally, the plasma levels of anthocyanidins after consuming red wine, elderberry extract, and blackcurrant juice are around 0.01 µM, 0.10 µM, and 0.11 µM, respectively [13,61,62].

The highest concentration of plasma flavonoids in humans usually occurs 1 to 2 h after the consumption of flavonoid-rich foods [36]. However, the level depends on the type of flavonoid, as anthocyanins and catechins have a half-life elimination that is 5 to 10 times less than that of flavonols [55]. Although data on the concentration of flavonoids in human tissue are scarce, flavonoids have been shown to play an important role in antioxidant defence in both cells and tissues [13].

## 6. Worldwide Flavonoid Intake

**Figure 4:**
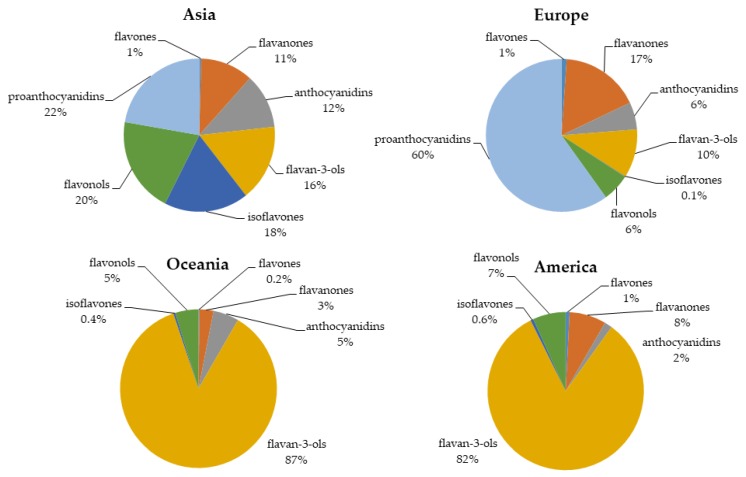
Worldwide intake of flavonoid subclasses.

The intake of flavonoids depends not only on the food itself and its bioavailability but also on geography, agricultural practices, climate stress, and cultural factors. Diets may differ in different locations [63]. Therefore, based on food frequency questionnaires (FFQs) administered in different studies, we extracted the following distribution of flavonoid consumption around the world (Figure 4).

### 6.1. Asia

A study performed by Ying Zhang et al. (2010) in China investigated the main sources of flavonoids in adults [64]. The mean intakes of total flavonoids, flavones, and flavonols were 19.13, 4.19, and 13.38 mg/day, respectively. The total intake of flavones and flavonols was attributable to fruits and vegetables. The main sources of flavonoids were *Actinidia* (5%), eggplant (7%), celery (7%), potato (8%), and apple (12%) [64]. Similarly, another study on female adolescents of northern China observed that the mean total flavonoid intake was 20.60 mg/day, with flavone and flavonol intakes of 4.31 and 16.29 mg/day, respectively. From lowest to highest, the food sources of flavonoids were aubergine (3.9%), leeks (3.9%), soybean sprouts (4.2%), celery (4.2%), tomatoes (4.2%), Chinese cabbage (4.7%), oranges (7%), lettuce (7.3%), potatoes (9.9%), and apple (11.7%) [65].

The major sources of dietary flavonoids in Korean adults were identified in a study performed by You Kin Kim et al. (2015) [66]. In this study, they observed that the total daily flavonoid intake was 107 mg/day and that the anthocyanidin, flavan-3-ol, flavanone, flavone, flavonol, and isoflavone intakes were 24.3, 21.8, 8.81, 0.97, 27.8, and 24.3 mg/day, respectively. The main food sources of flavonoids were, in ascending order, tangerine, radish, tofu, onion, soybeans, persimmons, green tea, and Kimchi (traditional fermented vegetable product) [66]. Another study performed by Shinyoung Jun et al. (2015) evaluated the dietary flavonoid intake in Korean adults (33,581 subjects, aged 19 years and older) [67]. The mean total flavonoid intake was 318 mg/day. The intakes of flavonoid subclasses were, in ascending order, flavones (0.4%), flavanones (11.3%), anthocyanidins (11.6%), flavan-3-ols (16.2%), isoflavones (18.1%), flavonols (20.3%), and proanthocyanidins (22.3%) [67].

### 6.2. Europe

Diets can differ according to region. Hence, Europe can be divided into southern and northern diets, known as the Mediterranean and Non-Mediterranean (UK and Nordic) diets, respectively.

The Mediterranean diet has been closely studied. Therefore, there is a large amount of data on Mediterranean nutrition. Among these studies, the European Prospective Investigation into Cancer and Nutrition Study (EPIC) is one of the most important. This study included 477,312 subjects from different countries in Europe, aged 35 to 70 years. For the Spanish cohort (40,683 subjects) in 2010, the mean total flavonoid intake was 313.26 mg/day [68]. From lowest to highest, the flavonoid subclass intakes were isoflavones (<0.01%), flavones (1.1%), anthocyanidins (5.8%), flavonols (5.9%), flavan-3-ols (10.3%), flavanones (16.9%), and proanthocyanidins (60.1%). The main dietary food sources of flavonoids were tea (2.2%), chocolate (2.6%), peaches (3.3%), pears (4%), beans (4.9%), oranges (9.3%), red wine (21%), and apples (23%) [68]. The main sources of proanthocyanidins were apples, red wines, and beans. Similarly, the most abundant sources of flavan-3-ols were, in ascending order, some fruits (such as plums, grapes, apricots, pears, and peaches), chocolate, apples, tea, and red wine. However, the most abundant sources of flavanones were citrus fruits and their derived products (such as juices) [69]. In the European region, the main food sources of anthocyanidin were fruits (such as pears, apples, and grapes), seeds, and nuts. These were followed by wine, isotonic drinks (in the Northern region), juices (Central region), and vegetables [70].

Another study performed by EPIC (36037 subjects aged 35–74 years) demonstrated that there are differences in the in flavonoid intake between European countries (Norway, Sweden, Denmark, the UK, the Netherlands, Germany, France, Italy, Spain, and Greece) [71]. The daily proanthocyanidin intake was the lowest in Greece and the highest in Spain. In contrast, the lowest intake of flavan-3-ols was observed in Greek women and men (124.8 and 160.5 mg/day, respectively), and the highest total intake was observed in women of the UK General population cohort (377.6 mg/day) and health-conscious men (453.6 mg/day). Likewise, flavan-3-ol monomer intake was the lowest in Greece (20.7 and 26.6 mg/day in women and men, respectively) and the highest in the UK general population (178.6 and 213.5 mg/day in women and men, respectively) [71]. The most important sources of flavan-3-ols in Mediterranean countries, non-Mediterranean countries, and the UK are non-citrus fruit, mainly apples, followed by wine and tea. Tea is responsible for the high flavan-3-ol intake in the UK [71]. For proanthocyanidins, the most important sources in Mediterranean countries are non-citrus fruits, and those in the UK are tea, wine, puddings, and pulses. However, in non-Mediterranean countries, the most important sources are non-citrus fruits, wine, and chocolate [71].

A study performed by Anna Vogiatzoglou et al. (2015) identified the main sources of flavonoids in the European Union [64]. The mean intake of total flavonoids was 428 mg/day, with the lowest intake in the Southern Region (301 mg/day), followed by the Northern Region (348 mg/day), and the highest intake was in the Central Region (506 mg/day), with flavan-3-ols the main flavonoid subclass consumed. Except for flavones and anthocyanidins (which had the highest intakes in the Northern Region), the highest intakes of all other flavonoid subclasses were in the Central Region. Regarding flavonoid sources, in the Southern region, the main sources of flavonoids were fruits and fruit products (mainly pome fruits and berries), but in the Northern and Central regions, tea was the main source of total flavonoids [72]. There were many regional differences, and in the Northern region, the intakes of flavanones and anthocyanidins were the highest, mainly in Finland where the primary sources are citrus fruits and berries, respectively. Nevertheless, in the Southern region, France had the highest intake of anthocyanidins and flavan-3-ols. This study also reported that Germany and Belgium had very low intakes of flavonoid-rich foods [72].

### 6.3. Oceania

A study that estimated the flavonoid intake in the Australian population (13,858 participants) obtained an average total flavonoid intake of 351 mg/day (of which 75% was flavan-3-ols and 15% was flavanones). In ascending order, the most important flavonoids sources were stalk vegetables, leaf, apples, wine, grapes, oranges, and black tea (which provided 76% of the flavonoid intake) [73]. In the Australian diet, the predominant sources of flavonols and flavon-3-ols were green and black tea as well as pears, apples, and wine for the latter [74]. Other significant sources of flavonols were beans, grapes, apple, broccoli, and onion. Wine was the main source of anthocyanidin. The main sources of flavone and flavanone were spinach and oranges, respectively [74]. However, the most recent Australian population study performed by Murphy KJ et al. (2019) reported an average total flavonoid intake of 660 and 566 mg/day for women and men, respectively [75]. In ascending order, the contributions to total flavonoids intake by subclass were flavones (0.2%), isoflavones (0.4%), flavanones (2.9%), flavonols (4.8%), anthocyanidins (5.3%), and flavan-3-ols (86.5%) [75]. Regarding the dietary sources of flavonoids, tea was responsible for 85% of the total flavonoid intake, followed by fruit juice (2.4%), apple (2.2%), wine (1.7%), berries (1.6%), banana (1.1%), cocoa (0.6%), citrus fruit (0.6%), plum (0.4%), grapes (0.4%), and nuts (0.4%) [75].

### 6.4. North America

In America, diets differ depending on the region. In North America, processed foods predominate in diets, whereas in South America, fruits and vegetables are the main components of the diet [76]. A study performed by Monica L Bertoia et al. (2016) analysed the dietary flavonoid intake of three prospective cohorts in United States, finding estimated averages of 236 mg/day and 224 mg/day for women and men, respectively [77]. Another prospective study in the United States estimated the intake of flavonoid subclasses, in increasing order, as isoflavones (0.6%), flavones (0.8%), anthocyanidins (1.6%), flavonols (6.8%), flavanones (7.6%), and flavan-3-ols (82.5%) [78]. Thus, the major dietary flavonoid sources were citrus fruits, wine, citrus fruit juices, and tea. In fact, tea was the main source for flavan-3-ols and flavonols [78]. Another study performed by Kim K et al. (2016) estimated the intake and major food sources of flavonoids in adults in the United States [79]. The major dietary sources were apples, wine, citrus fruit, berries, citrus fruit juices, and tea, with tea as the major contributor of flavan-3-ols and flavonols, at 155.9 and 164.4 mg/day, respectively, of the total flavonoids [79].

For Europe and the United States, numerous descriptive studies on flavonoid intake have been published, but for Latin-American countries, there are insufficient data available. However, *Raul* Zamora-Ros et al. (2018) analysed polyphenol dietary intake in the Mexican Teachers’ Cohort, reporting an average total flavonoid intake of 235 mg/day. In this population, the main food sources of total polyphenols were orange juice (4.8%), mandarins (5.1%), apples (7.2%), and coffee (47.4%) [80].

There are some differences between the intake of flavonoid subclasses around the world (Table 9). However, it is unclear if these differences are related to differences in cancer incidence. To clarify this issue, for this review, the latest epidemiological studies and GLOBOCAN data (2018) [2] were collected.

## 7. Antioxidant Activity of Dietary Flavonoids and Cancer Incidence

All biological processes in an organism must remain in homeostasis. When the pro-oxidant load and antioxidant defence are unbalanced, reactive oxygen species (ROS) are produced, and free radicals are generated [81]. Oxidative stress is characterised by the amount of ROS produced and is closely related to development of some diseases such as cancer caused by oxidative lesions in DNA. However, there are other mechanisms that protect organisms against oxidation, including good nutrition [81]. Thus, the interest in finding compounds with antioxidant activity such as flavonoids has increased. Among them, apigenin (a plant-derived food polyphenol, with sources such as chamomile tea and celery) seems to have strong antioxidant activity in neurological disorders [82]. Myricitrin has been isolated from Daebong persimmon peel, and this flavonoid has strong antioxidant activity through its ferric ion reducing antioxidant ability [83]. Another flavonoid, hesperetin, was shown to ameliorate oxidative stress in disease conditions such as dyslipidaemia and hyperglycaemia in a murine model [84]. In addition, in diabetic rats, galangin reduced hyperglycaemia-mediated oxidative stress and improved the antioxidant status [85]. Under abnormal conditions such as hyperammonemia in rats, quercetin was found to protect against oxidative stress and exert anti-inflammatory activity [86]. During induced oxidative stress in rats, rutin was found to act as a strong antioxidant protecting against oxidative effects [87]. Moreover, in another in vitro study, it was demonstrated that kaempferol has moderate oxygen radical absorption capacity and strong radical-scavenging activity [80]. In murine tissues, quercetin protects against induced oxidative damage [88]. Several studies have investigated the antioxidant activity of flavonoids in humans [89]. Among them, a study performed by Alipour B. et al. (2016) suggested an association between serum total antioxidant capacity and total flavonoid consumption [90]. However, they attributed antioxidant activity to anthocyanins [90]. Thus, there is evidence indicating the strong antioxidant activity of flavonoids in vitro and in vivo, and many epidemiological studies have shown that dietary flavonoids are associated with a lower incidence of cancer. Therefore, because cancer is a major health problem worldwide, it would be of value to determine if its incidence is associated with dietary flavonoid intake and what intake amount would reduce cancer risk.

**Figure 5:**
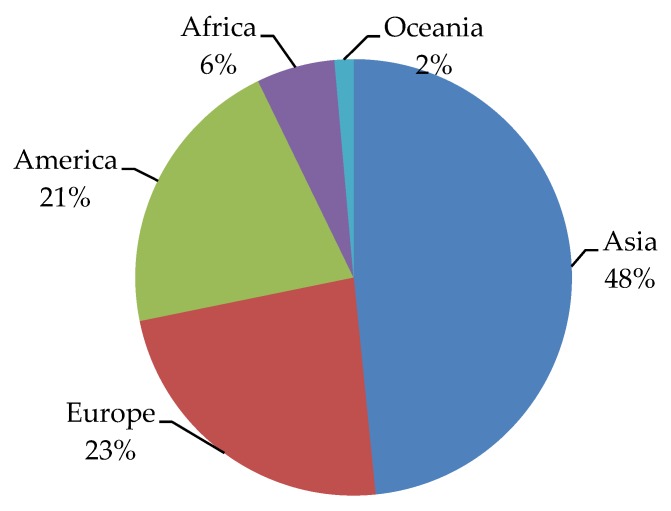
Worldwide cancer incidence. Data collected from GLOBOCAN (2018) [2].

The latest data collected from GLOBOCAN [2] indicate differences in total cancer incidence around the world. Asia is responsible for 48% of the total cancer incidence (Figure 5).

**Figure 6:**
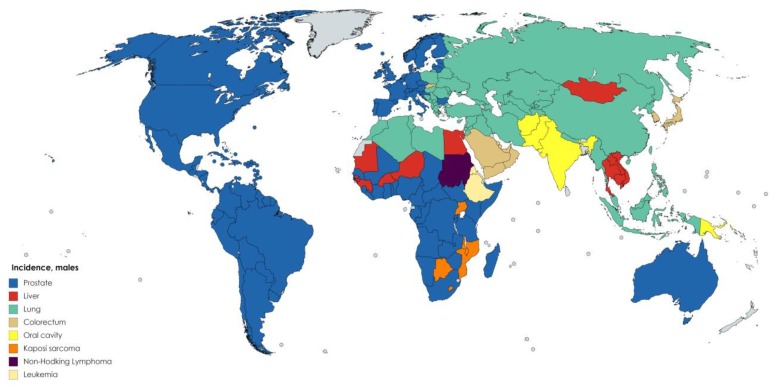
Worldwide cancer incidence by type in males [2]. Created with mapchart.net.

**Figure 7:**
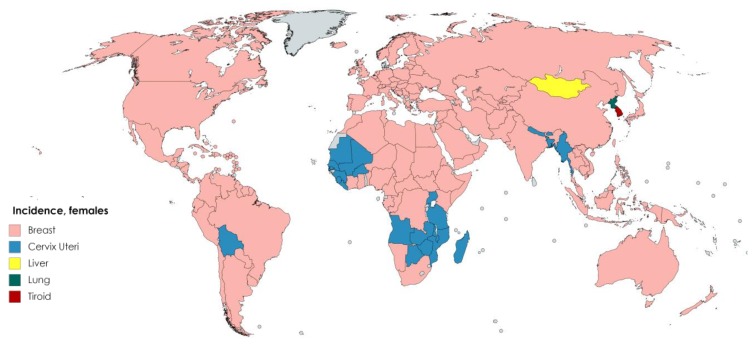
Worldwide cancer incidence by type in females [2]. Created with mapchart.net.

However, incidence can vary according to gender for different types of cancer around the world. According to GLOBOCAN [2] data, the cancer types with the highest incidence in males are, in decreasing order, lung, prostate, stomach, liver, and colorectal cancers (Figure 6). For females, breast cancer accounts for the highest number of cancer cases, followed by lung cancer (Figure 7).

Regarding breast cancer (Table 10), in the European Prospective Investigation into Cancer and Nutrition (EPIC) study, flavonoid dietary intake and breast cancer risk were analysed in a cohort of 334,850 women with an 11.5 year follow up [91]. Within this cohort were 11,576 breast cancer cases. However, there was no statistically significant association between total flavonoid (Hazard Ratio (HR) 0.97, 95% Confidence Interval (CI): 0.90–1.07) and isoflavone (HR 1, 95%CI: 0.91–1.10) intakes and breast cancer risk [91]. Another prospective study evaluated coffee and tea intake and its relationship with breast cancer risk in black women [92]. The results showed that among the 52.062 participants, there were 1268 incident cases of breast cancer during 12 years of follow up. The data showed that that the intake of coffee (Internal Rate of Return (IRR): 1.03, 95% CI: 0.77–1.39) or tea (IRR: 1.13, 95% CI: 0.78–1.63) was not associated with the risk of breast cancer [92]. Regarding tea and coffee intake, a study performed in Sweden suggested that tea intake is positively associated with oestrogen and progesterone receptor-positive breast cancer, but that coffee consumption is negatively associated with the risk of oestrogen receptor-positive, progesterone receptor-negative breast cancer [93]. Another study performed in Shanghai attempted to associate urinary polyphenols with breast cancer risk [94]. They measured tea flavonols (kaempferol and quercetin) and polyphenols as epicatechin in a cohort with 353 cases and 701 controls. They observed an inverse association between breast cancer risk and urinary excretion of epicatechin (Odds Ratio (OR) 0.59, 95% CI: 0.39–0.88) [94]. Thus, it was concluded that epicatechin-rich foods could reduce breast cancer risk.

There is controversy regarding the association between breast cancer and isoflavone intake because of its possible role in oestrogen metabolism. Thus, a case-control study in south-western China investigated the relationship between oestrogen metabolism, soy isoflavones, and breast cancer risk [95]. The findings suggested a protective effect of a high soy isoflavone intake on breast cancer risk based on the relation of oestrogen metabolites and breast cancer [95]. Furthermore, regarding other flavonoid subclasses, a meta-analysis of epidemiologic studies performed in 2013 demonstrated that breast cancer risk had a direct association with flavone (Relative Risk (RR): 0.83, 95% CI: 0.76–0.91) and flavonol intake in women (RR: 0.88, 95% IC: 0.80–0.98) [96]. Likewise, in another study performed by Cutler et al. (2008) that analysed cancer risk in postmenopausal women in relation with dietary flavonoid intake, an inverse association was obtained between isoflavone intake and cancer incidence (HR: 0.93, 95% CI: 0.86–1.00), and an inverse association was found between proanthocyanidin (HR: 0.75, 95% CI: 0.57–0.97) and flavanone (HR: 0.68, 95% CI: 0.53–0.86) intake and lung cancer incidence [14].

Regarding total polyphenol intake and breast cancer risk, a study performed by Gardeazabal et al. (2018) that included more than 22,000 Spanish university graduates showed that menopausal status is an important factor in breast cancer risk [97]. Thus, they found no significant association between breast cancer risk and total polyphenol intake. However, in postmenopausal women, they observed an inverse association between breast cancer risk and total polyphenol intake (HR: 0.31, 95% CI: 0.13–0.77) [97]. Because they have antioxidant activity and similar chemical compositions as oestrogens, flavonoids are able to reduce menopause symptoms [98]. However, further research is needed to demonstrate the effect of flavonoid intake on pre- and post-menopause breast cancer risk.

A case-control study performed by Christensen et al. (2012) (Table 10), which analysed the association of lung cancer risk with flavonoid intake, did not find an association between flavonoid intake and risk reduction. However, a low intake of total and different subclasses of flavonoids was related to an increased risk of lung cancer. The ORs (95%CI) were 0.63 (0.47–0.85) for total flavonoids, 0.70 (0.53–0.94) for flavanones, 0.62 (0.45–0.84) for flavonols, 0.68 (0.50–0.93) for flavones, 0.67 (0.50–0.90) for flavan-3-ols, and 0.82 (0.61–1.11) for anthocyanidins [99].

A population-based case-control study carried out on a population of Sicilian men analysed the association between dietary factors, such as flavonoids, and prostate cancer risk (Table 10). The results suggested that prostate cancer risk could be reduced by a high intake of catechins (OR: 0.12, 95% CI: 0.04–0.36) and flavonol (OR: 0.19, 95% CI: 0.06–0.56). However, the risk seemed to increase with a high intake of flavanones [100].

Gastric cancer is the second main cause of cancer deaths and the fourth most common cancer worldwide (Table 11) [68]. In a case-control study performed in Korea, a significant association was found between total flavonoid intake and gastric cancer risk reduction in women (OR 0.33, 95% CI 0.15–0.73) but not in men [101]. Furthermore, the EPIC study investigated the association between gastric adenocarcinoma risk and flavonoid intake [68]. They observed an inverse association between gastric adenocarcinoma risk and total flavonoid intake in women (HR 0.81, 95% CI 0.70, 0.94). This association was observed for some flavonoid subclasses such as flavanols, flavones, flavonols, and anthocyanidins [68]. However, in a prospective study carried out in the United States that analysed all cancers, researchers observed that flavonoid intake was associated with protection against neck and head cancer risk but not gastric cancer risk [102].

Pancreatic cancer has the worst prognosis of all cancers (Table 11), and its mortality/incidence ratio is 0.98 [96]. However, a study performed on the EPIC cohort examined the association between pancreatic cancer risk and flavonoid intake and found an inverse association between them, although it was not statistically significant [103].

Regarding colorectal cancer (Table 11), it has been demonstrated that flavonoids are able to inhibit the growth of colon cancer cells in vitro [104]. However, in human-based studies, the results are different. In a prospective study that examined daily flavonoid intake and its relationship with colorectal cancer, the data showed there was no association between the risk of colorectal cancer and flavonoid intake [104]. Important results were obtained in a study performed by Xu M. et al. (2016). Their data showed that there was an inverse association between flavonoid intake as anthocyanidins, flavanones, and flavones and colorectal cancer risk. However, this only occurred when the sources of flavonoids were fruits and vegetables [105]. A case-control study performed on a Spanish population found an inverse association between the risk of colorectal cancer and intake of total flavonoids (OR: 0.59, 95% CI, 0.35–0.99) and some flavonoid subclasses (such as proanthocyanidins and flavones) [106]. The same researchers performed a case-control study to analyse the relationship between flavonoid intake and colorectal cancer recurrence and survival. However, their results did not support the beneficial effects of flavonoids on colorectal cancer prognosis [107].

## 8. Conclusions

In summary, there remains controversy regarding the possible protective effect of flavonoids on cancer in epidemiological studies. However, this association could vary depending on many factors such as geographical location and diet. It appears that some flavonoid subclasses suggest a decrease of the risk of different types of cancer, such as catechin and flavonols for prostate cancer, epicatechin for breast cancer, proanthocyanidins for lung cancer, flavones for colorectal cancer, and total flavonoids for gastric cancer. Thus, because the main sources of these flavonoids are different, the risk of cancer could be reduced by including them in a healthy diet, which would be mainly based on vegetables and fruits, whole grain cereals, legumes, seeds, and nuts, as well as cocoa, coffee, fruit juices, and tea. However, further studies are needed to investigate and confirm this hypothesis that a healthy diet can help decrease the incidence of different types of cancer.

## Figures and Tables

**Table 1:** Flavonoid contents of fruits (mg/100 g food). Data collected from Phenol Explorer [24].

Fruits	ANT	DYC	FVA	FVO	Total
Berries					
Aestivalis grape	79.74	-	-	1.7	81.44
American cranberry	49.89	-	-	43.84	93.73
Black chokeberry	878.11	-	-	134.87	1012.98
Black elderberry	1316.66	-	-	42	1358.66
Black raspberry	-	-	-	19	19
Blackberry	172.59	-	13.87	16.87	203.33
Blackcurrant	593.58	-	1.17	13.68	608.43
Black grape	72.15	-	14.03	4.01	90.19
Green grape	-	-	3.78	2.49	6.27
Green currant	-	-	-	11.07	11.07
Highbush blueberry	156.6	-	1.11	54.77	212.48
Lingonberry	60.21	-	-	48.98	109.19
Lowbush blueberry	204.56	-	-	-	204.56
Red raspberry	72.47	-	5.73	16.26	94.46
Redcurrant	33.13	-	4.68	0.77	38.58
Strawberry	26.87	-	9.1075	2.32	38.29
**Drupes**					
Nectarine	0.86	-	17.65	1.35	19.86
Peach	0.28	-	45.18	1.42	46.88
Plum	47.79	-	46.9	6.98	101.67
Sour cherry	54.43	-	0.2	-	54.63
Sweet cherry	170.18	-	14.87	-	185.05
**Pomes**					
Apple	0.93	5.38	39.42	10.62	56.35
Pear	-	-	4.98	0.84	5.82
Quince	-	-	7.49	0.67	8.16
**Tropical Fruits**					
Banana	-	-	1.55	-	1.55
Kiwi	-	-	0.7	-	0.7
Mango	-	-	1.72	-	1.72
Persimmon	-	-	1.28	-	1.28
Pomegranate	-	-	1.1	-	1.1

ANT: Anthocyanins, DYC: Dihydrochalcones, FVA: Flavan-3-ols, FVO: Flavonols.

**Table 2:** Flavonoid contents of vegetables (mg/100 g food) [24].

Vegetables	ANT	CHA	FVA	FNE	FVE	FVO	Total
Cabbages							
Broccoli	-	-	-	-	-	27.8	27.8
**Fruit Vegetables**							
Avocado	-	-	0.55	-	-	-	0.55
Black olive	82.97	-	-	-	27.43	49.43	159.83
Green olive	-	-	-	-	0.56	-	0.56
Green sweet pepper	-	-	-	-	2.11	5.49	7.6
Red sweet pepper	-	-	-	-	0.05	0.24	0.29
Tomato	-	-	-	0.14	-	0.014	0.15
**Leaf Vegetables**							
Curly	-	-	-	-	-	24.06	24.06
Escarole	-	-	-	-	-	18.23	18.23
Green lettuce	-	-	-	-	0.4	3.99	4.39
Red lettuce	3.53		-	-	2.51	16.74	22.78
Spinach	-	-	-	-	-	119.27	119.27
**Onion-Family Vegetables**							
Red onion	9	-	-	-	-	122.51	131.51
White onion	-	-	-	-	-	5.4	5.4
Yellow onion	-	-	-	-	-	59.1	59.1
Shallot	-	-	-	-	-	112.22	112.22
**Pod Vegetables**							
Broad bean pod	-	0.08	154.45	-	0.37	34.64	189.54
Green bean	-	-	2.42	-	-	5.55	7.97
**Shoot Vegetables**							
Asparagus	-	-	-	-	-	23.19	23.19
Globe artichoke, heads	-	-	-	-	57.8	-	57.8

ANT: Anthocyanins, CHA: Chalcones, FVA: Flavan-3-ols, FNE: Flavanones, FVE: Flavones, FVO: Flavonols.

**Table 3:** Flavonoid contents of seeds (mg/100 g food) [24].

Seeds	ANT	FVA	FNE	FVE	FVO	IFA	Total
Nuts							
Almond	-	4.93	0.5	-	3.81	0.06	9.3
Cashew nut	-	1.1	-	-	-	-	1.1
Chestnut	-	0.05	-	-	-	-	0.05
Hazelnut	-	5.7	-	-	-	-	5.7
Peanut	-	-	-	-	-	0.51	0.51
Pecan nut	-	16.7	-	-	-	-	16.7
Pistachio		6.9	0.12	0.103	0.07		7.193
**Common Bean**							
Black common bean	41.05	-	-	-	10	1.4	52.45
Others common bean	7.42	-	-	-	69.58	0.2	77.2
White common bean	0.13	-	-	-	49.96	0.5	50.59
**Other Beans**							
Broad bean seed whole	-	49.37	-	-	-	-	49.37
Sunflower seed meal	-	-	-	-	-	0.02	0.02
**LENTILS**							
Lentils	-	5.17		0.95	1.09	-	7.21
**Soy Products**							
Soy paste miso	-	-	-	-	-	63.09	63.09
Soy tempeh	-	-	-	-	-	147.74	147.74
Soy tofu	-	-	-	-	-	39.24	39.24
Soybean roasted	-	-	-	-	-	253.11	253.11

ANT: Anthocyanins, FVA: Flavan-3-ols, FNE: Flavanones, FVE: Flavones, FVO: Flavonols, IFA: Isoflavonoids.

**Table 4:** Flavonoid contents of cereals (mg/100 g food) [24].

Cereals	FVA	FVE	FVO	Total
Cereals				
Barley, whole grain flour	35.2	-	-	35.2
Buckwheat groats, thermally treated	-	-	8.96	8.96
Buckwheat, refined flour	-	-	5.86	5.86
Buckwheat, whole grain flour	-	0.9	36.14	37.04
Common wheat, refined flour	-	18.4	0.08	18.48
Common wheat, whole grain flour	-	77.29	0.11	77.4

FVA: Flavan-3-ols, FVE: Flavones, FVO: Flavonols.

**Table 5:** Flavonoid contents of cocoa (mg/100 g food) [24].

Cocoa	FVA	FVO	Total
Cocoa			
Chocolate dark	212.36	25	237.36
Chocolate milk	19.22	-	19.22
Cocoa powder	511.62	-	511.62

FVA: Flavan-3-ols, FVO: Flavonols.

**Table 6:** Flavonoid contents of oils (mg/100 g oil) [24].

Oils	FVE	Total
Oils		
Extra virgin olive oil	1.53	1.53
Virgin olive oil	0.23	0.23
Refined olive oil	0.15	0.15

FVE: Flavones.

**Table 7:** Flavonoid contents of non-alcoholic beverages (mg/100 g drink) [24].

Non-Alcoholic Beverages	ANT	DYC	FVA	FNE	FVE	FVO	IFA	Total
Cocoa Beverage								
Chocolate, milk	-	-	20.33	-	-	-	-	20.33
**Fruit Juices**								
**Berry Juices**								
Fox grape juice	-	-	5.9	-	-	-	-	5.9
Green grape juice	-	-	3.88	-	-	-	-	3.88
Grapefruit juice	-	-	-	46.44	-	0.68	-	47.12
**Citrus Juices**								
Lemon juice	-	-	-	32.66	4.77	-	-	37.43
Lime juice	-	-	-	19.61	-	-	-	19.61
Orange juice	3.17	-	-	37.63	6.14	1.08	-	48.02
Pummelo juice	-	-	-	8.48	-	-	-	8.48
Red raspberry juice	-	-	-	-	-	9.58	-	9.58
Rowanberry	-	-	-	-	-	7.04	-	7.04
**Drupe Juices**								
Plum juice	-	5.85	24.7	-	-	-	-	30.55
**Pome Juices**								
Apple juice	-	4.39	48.45	-	-	2.15	-	54.99
Apple (cider) juice	-	4.78	22.66	-	-	-	-	27.44
Pear juice	-	-	3.24	-	-	-	-	3.24
**Tropical Juices**								
Kiwi juice	-	-	0.38	-	-	0.09	-	0.47
Pomegranate juice	10.13	0.1	-	-	-	0.25	-	10.48
**Herb Infusions**								
German chamomile, tea	-	-	2.07	-	-	-	-	2.07
Lemon verbena	-	-	10.6	-	-	-	-	10.6
Peppermint, tea	-	-	10.23	-	-	-	-	10.23
**Tea Infusion**								
Fennel tea	-	-	-	-	-	3.26	-	3.26
Black tea	-	-	73.29	-	-	10.06	-	83.35
Green tea	-	-	71.18	-	-	6.26	-	77.44
Oolong tea	-	-	35.72	-	-	-	-	35.72
**Soy Products**								
Soy milk	-	-	-	-	-	-	18	18

ANT: Anthocyanins, DYC: Dihydrochalcones, FVA: Flavanols, FNE: Flavanones, FVE: Flavones, FVO: Flavonols, IFA: Isoflavonoids.

**Table 8:** Flavonoid contents of alcoholic beverages (mg/100 g drink and mg/100 mL wine) [24].

Alcoholic Beverages	ANT	DYC	DYF	FVA	FNE	FVE	FVO	IFA	Total
Beer									
Beer (alcohol free)	-	0.0003	-	0.11	0.01	-	-	-	0.12
Beer (ale)	-	0.01	-	0.38	0.24	-	-	0.02	0.65
Beer (dark)	-	0.03	-	0.03	0.15	-	-	-	0.21
Beer (regular)	-	0.001	-	0.61	0.04	0.004	0.09	0.02	0.77
**Wines**									
Red wine	23.3	-	5.44	47.02	0.85	-	7.35	-	83.96
Rosé wine	-	-	0.38	2	-	-	-	-	2.38
White wine	0.04	-	0.57	2.07	0.23	-	0.695	-	3.61

ANT: Anthocyanins, DYC: Dihydrochalcones, DYF: Dihydroflavonols, FVA: Flavan-3-ols, FNE: Flavanones, FVE: Flavones, FVO: Flavonols, IFA: Isoflavonoids.

**Table 9:** Flavonoids intake and main food sources worldwide.

Country	Intake (mg/d)	Subclass	Food Sources
Asia	107	Protanthocyanidins > flavonols > isoflavons > flavan-3-ols > anthocyanidins > flavanones > flavones	Kimchi, green tea, persimmons, soybeans, onions
Southern europe	313	Proanthocyanidins > flavanones > flavan-3-ols > flavonols > anthocyanidins > flavones > isoflavones	Apples, red wine, oranges, beans, pears, peaches
Northern europe	348	Flavan-3-ols > flavones > anthocyanidins > flavonols > flavanones > isoflavones	Tea, citrus fruits, berries
Central europe	506	Flavan-3-ols > anthocyanidins > proanthocyanidins > flavanones > flavonols > flavones > isoflavones	Tea, non-citrus fruits, wine
Oceania	351	Flavan-3-ols > anthocyanidins > flavonols > flavanones > isoflavones > flavones	Black tea, oranges, grapes, wine, apples
North America	230	Flavan-3-ols > flavanones > flavonols > anthocyanidins > flavones > isoflavones	Apples, wine, citrus fruit juices and tea.

**Table 10:** Association between flavonoid intake and risk of breast, lung, and prostate cancers.

Authors	Methods	Results
Breast		
Itziar Gardeazabal et al. (2018) [97]	Prospective Cohort Study 10,713 Spanish Women Food Frequency Questionnaire (FFQ) Phenol-Explorer database HPLC	There was not a statistically significant association between total flavonoids and breast cancer risk. However, in postmenopausal women, the data indicate an inverse association between breast risk cancer and total polyphenol intake (HR: 0.31, 95% CI: 0.13–0.77)
Oh, J.K et al. (2015) [93]	Prospective Cohort Study 42,099 Swedish Women 30–49 years FFQ	Data showed that compared with no consumption, women who consumed >1 cup tea/day had an increased breast cancer risk (Relative Risk (RR): 1.19, 95% Confidence Interval (CI): 1.00–1.42), but women with a high intake of coffee (3–4 cups/day) had a decreased breast cancer risk (RR: 0.87, 95% CI: 0.76–1.00).
Raul Zamora-Ros et al. (2013) [91]	Prospective Cohort Study 334,850 women, 35–70 years 11.5 years follow up FFQ Phenol-Explorer database	There was no statistically significant association between total flavonoid (Hazard Ratio (HR) 0.97, 95% CI: 0.90–1.07) and isoflavone (HR 1, 95%CI: 0.91–1.10) intake and breast cancer risk.
Wang et al. (2011) [95]	Case-Control Study. 400 cases and 400 controls. Daily intake of soy isoflavones data Gene sequencing	They suggested a protective role of high soy isoflavone intake against breast cancer risk based on the relation of oestrogen metabolites, breast cancer, and isoflavone metabolism.
Boggs et al. (2010) [92]	Prospective Cohort Study 52,062 women, 21–69 years 12 years follow up FFQ	Data showed that the intake of coffee (Internal Rate of Return (IRR): 1.03, 95% CI: 0.77–1.39) or tea (IRR: 1.13, 95% CI: 0.78–1.63) was not associated with risk of breast cancer in participants.
Luo JF et al. (2010) [94]	Case-Control Study 353 cases, 701 controls, 40–70 years FFQ Liquid chromatography	There was an inverse association between breast cancer risk and urinary excretion of epicatechin (Odds Ratio (OR) 0.59, 95% CI: 0.39–0.88).
**Lung**		
Christensen KY et al. (2012) [99]	Case-Control Study 1061 cases and 1425 controls FFQ	A low intake of flavonoids was related to an increased risk of lung cancer. OR: 0.63, 95% CI: 0.47–0.85.
Cutler et al. (2008) [14]	Prospective Cohort Study USDA database FFQ	There was an inverse association between isoflavone intake and cancer incidence (HR: 0.93, 95% CI: 0.86–1.00) and an inverse association between proanthocyanidin (HR: 0.75, 95% CI: 0.57–0.97) and flavanone (HR: 0.68, 95% CI: 0.53–0.86) intake with lung cancer incidence.
**Prostate**		
Giulio Reale et al. (2018) [100]	Case-Control study 118 cases and 222 controls FFQ Prostate Specific Antigen	High intake of some subclasses of flavonoids (catechin (OR: 0.12, 95% CI: 0.04–0.36) and flavonol (OR: 0.19, 95% CI: 0.06–0.56) significantly reduces the risk of prostate cancer.

**Table 11:** Association between flavonoid intake and risk of gastric, pancreatic, and colorectal cancers.

Authors, Year	Methods	Results
Gastric Adenocarcinoma		
Sun L et al. (2017) [102]	Prospective cohort study 469,008 participants 12 years follow up FFQ	Data suggested that total flavonoid intake was associated with a reduced risk of neck and head cancer (HR: 0.76, 95% CI: 0.66–0.86).
Hae Donw Woo et al. (2014) [101]	Case-Control study. 334 cases and 334 controls, aged 35–75 years, from Korea. FFQ USDA database	Total flavonoids and their subclasses were significantly associated with a reduced risk of gastric cancer in women (OR 0.33, 95% CI 0.15–0.73) but not in men.
Zamora-Ros et al. (2012) [68]	Observational study 477,312 subjects, aged 35–70 years, from 10 European countries. Average Follow-up of 11 years FFQ USDA and Phenol Explorer Databases.	Total dietary intake was associated with a significant reduction in the risk of gastric adenocarcinoma in women (HR 0.81, 95% CI 0.70, 0.94).
**Pancreatic Cancer**		
Molina-Montes E. et al. (2016) [103]	Prospective cohort 477,309 participants Average Follow-up of 11 years FFQ USDA and Phenol Explorer Databases.	There was no association with pancreatic cancer risk and dietary flavonoid intake (HR: 1.09, 95% CI: 0.95–1.11); however, there was an inverse association, but not statistically significant, between prostate cancer risk and flavanone intake.
**Colorectal Cancer**		
Nimptsch K et al. (2016) [104]	Prospective cohort 42,478 male 76,364 female 26 years follow up FFQ	Data did not show an association between colorectal cancer risk and flavonoid subclass intake. RR (95% IC) values were 0.98 (0.81, 1.19) for anthocyanins, 1.07 (0.95, 1.21) for flavan-3-ols, 0.96 (0.84, 1.10) for flavanones, 1.01 (0.89, 1.15) for flavones, and 1.04 (0.91, 1.18) for flavonols.
Xu M. et al. (2016) [105]	Case-Control study 1632 cases and 1632 controls FFQ	There was an inverse association between colorectal cancer risk and flavone (OR: 0.54, 95% CI 0.43, 0·67), flavanone (OR: 0.28, 95% CI: 0.22–0.36), and anthocyanidin (OR: 0.80, 95% CI: 0.64–1.00) intake.
Zamora Ros et al. (2015) [107]	Case-control study 523 participants FFQ	Flavonoid intake was not associated with colorectal cancer survival or recurrence.
Zamora Ros et al. (2013) [108]	Case-Control study 424 cases and 401 hospital-based controls FFQ Phenol Explorer Database	Data showed an inverse correlation between risk of colorectal cancer and intake of total flavonoids (OR: 0.59, 95% CI, 0.35–0.99) and some flavonoid subclasses (such as proanthocyanidins and flavones)
